# Prophylaktische Hirnbestrahlung mit und ohne Hippocampusschonung beim kleinzelligen Bronchialkarzinom (PREMER) – eine randomisierte Phase-III-Studie

**DOI:** 10.1007/s00066-021-01899-7

**Published:** 2022-01-13

**Authors:** Fabian Schunn, Stefan Koerber

**Affiliations:** Radiologische Klinik | Klinik für RadioOnkologie und Strahlentherapie, Universitätsklinikum Heidelberg, Im Neuenheimer Feld 400, 69120 Heidelberg, Deutschland

## Hintergrund der Arbeit

Die Höhe der Strahlendosis, der neuronale Stammzellen des Hippocampus im Rahmen einer Ganzhirnbestrahlung ausgesetzt sind, verschlechtert die Neurokognition. Die hauptsächliche Befürchtung bei der Durchführung einer prophylaktischen Hirnbestrahlung mit Hippocampusschonung (HA-PCI) bei Patienten mit kleinzelligem Bronchialkarzinom (SCLC) ist das Auftreten von Hirnmetastasen im Schonungsareal des Hippocampus.

## Methode und Patientengut

Diese Phase-III-Studie schloss insgesamt 150 SCLC-Patienten (71,3 % im Stadium „limited disease“) ein, die entweder eine prophylaktische Standardhirnbestrahlung (PCI; 25 Gy in 10 Fraktionen) oder eine HA-PCI erhielten. Primärer Endpunkt der Studie war der Delayed-free-recall-Score (DFR) des Free and Cued Selective Reminding Tests (FCSRT) drei Monate nach Bestrahlung. Eine Abnahme um drei oder mehr Punkte vom Ausgangswert wurde dabei als relevante Verschlechterung gewertet. Sekundäre Endpunkte waren die übrigen FCSRT-Scores, Lebensqualität (QoL), Inzidenz und Lokalisation von Hirnmetastasen sowie das Gesamtüberleben (OS). Die Daten wurden zu Beginn sowie 3, 6, 12 und 24 Monate nach der PCI erfasst.

## Ergebnisse

Die Patientencharakteristika waren zwischen den beiden Gruppen ausgeglichen. Der mediane Nachbeobachtungszeitraum lag bei den überlebenden Patienten bei 40,4 Monaten. Eine Abnahme des DFR-Scores drei Monate nach Therapie war im HA-PCI-Arm mit 5,8 % vergleichsweise seltener als im PCI-Arm mit 23,5 % (Odds Ratio 5; 95 %-CI 1,57–15,86; *p* = 0,003). Die Analyse aller FCSRT-Scores zeigte eine Abnahme des „total recall“ (TR; 8,7 % vs. 20,6 %) nach drei Monaten, DFR (11,1 % vs. 33,3 %), TR (20,3 % vs. 38,9 %) und „total free recall“ (14,8 % vs. 31,5 %) nach sechs Monaten und TR (14,2 % vs. 47,6 %) nach 24 Monaten. Die Inzidenz von Hirnmetastasen, das Gesamtüberleben und die Lebensqualität unterschieden sich nicht signifikant.

## Schlussfolgerung der Autoren

Die Schonung des Hippocampus während einer PCI verbessert den Erhalt der Neurokognition bei Patienten mit SCLC. Verglichen mit der Standard-PCI wurden keine Unterschiede bezüglich des zerebralen Therapieversagens, des Gesamtüberlebens und der Lebensqualität beobachtet.

## Kommentar

Die PCI war bei SCLC-Patienten lange Zeit nahezu unumstritten, nachdem Slotman et al. [2] 2007 eine signifikante Reduktion der Inzidenz von Hirnmetastasen sowie ein verlängertes Gesamtüberleben nach PCI aufzeigen konnten. Erst im weiteren Verlauf kamen Diskussionen bezüglich der Patientenselektion auf. Takahashi et al. [3] konnten im Gesamtüberleben von Patienten im Stadium „extensive disease“ keinen Vorteil der PCI gegenüber einer Kontrollgruppe finden, was zu einer strengeren Indikationsstellung beitrug. Heute wird die PCI überwiegend für Patienten im Stadium „limited disease“ empfohlen und unter dem Standpunkt möglicher Nebenwirkungen kritisch verfolgt [1].

Daten zur Neurotoxizität nach zerebraler Radiotherapie stammen bislang vor allem aus Kollektiven nach palliativer Ganzhirnbestrahlung, liegen vereinzelt aber auch bei Patienten nach PCI vor. Ein hohes Lebensalter, bereits vorbestehende neurokognitive Einschränkungen und die applizierte Dosis scheinen prädiktiv für eine Verschlechterung der Neurokognition nach Radiotherapie zu sein [4]. Vor allem im Setting der prophylaktischen Hirnbestrahlung ist die Vermeidung von Neurotoxizität für Patienten besonders relevant, sodass sich die Frage nach schonenderen Verfahren stellt. Bislang gehörte die Hippocampusschonung dabei nicht zur klinischen Routine – ein Umstand, dessen Veränderung die Autoren auf Basis der veröffentlichten Daten nun fordern.

Was macht den Hippocampus besonders schützenswert? Neben der subventrikulären Zone um die Seitenventrikel ist er nach aktuellem Wissensstand eines der wenigen Hirnareale, in denen beim erwachsenen Menschen Neurogenese stattfindet. Hierfür ist vorwiegend die proliferative Kapazität der neuronalen Progenitorzellen verantwortlich, welche sich in der subgranulären Zone des Gyrus dentatus befinden [5]. Eine radiogene Schädigung dieser Zellen trägt mutmaßlich zu einer Dysfunktion des Hippocampus mit Abnahme der Lernfähigkeit, Gedächtnisleistung und räumlichen Informationsverarbeitung bei.

In der vorliegenden Arbeit konnte die mittlere Hippocampusdosis unter PCI durch eine entsprechende Schonung von 24,5 Gy (SD 2,1 Gy) auf 10,9 Gy (SD 2,0 Gy) reduziert werden, was klinische Relevanz hinsichtlich des Erhalts der Neurokognition prinzipiell plausibel erscheinen lässt. Das Auftreten von Hirnmetastasen nach zwei Jahren unterschied sich mit 22,8 % in der HA-PCI- und 17,7 % in der PCI-Gruppe nicht signifikant. Ebenso zeigte sich kein Nachteil im medianen Gesamtüberleben, welches bei 23,4 Monaten (HA-PCI) bzw. 24,9 Monaten (PCI) lag. Diese Ergebnisse unterstreichen, wie die ebenfalls dieses Jahr veröffentlichten Daten von Belderbos et al. [6], dass die Hippocampusschonung im Rahmen der PCI wohl sicher durchführbar ist.

Die Kollektive der beiden Studien schlossen mit rund 70 % überwiegend Patienten im Stadium „limited disease“ ein. Interessanterweise konnte in den holländischen Daten jedoch kein positiver Einfluss der Hippocampusschonung auf den Erhalt der Neurokognition festgestellt werden. Ursächlich hierfür könnte in erster Linie die jeweilige Messmethodik sein. Während in der holländischen Studie der Hopkins Verbal Learning Test (HVLT) verwendet wurde, griffen Rodriguez et al. auf den FCSRT zurück. Die Unterschiede sowie der vermeintliche Vorteil des FCSRT in der Diagnostik hippocampusspezifischer Defizite werden von den Autoren ausgiebig diskutiert. Zusammenfassend werden diesbezüglich ein vermuteter Deckeneffekt des HVLT, das Fehlen einer Interferenzphase und eine stärkere Beeinflussbarkeit durch mangelnde Aufmerksamkeit in der Lernphase angeführt.

Als primärer Endpunkt der Arbeit wurde eine Verschlechterung im DFR-Score des FCSRT um mindestens eine Standardabweichung drei Monate nach Radiotherapie definiert. Zu diesem Zeitpunkt wurde ein signifikanter Unterschied zugunsten der HA-PCI-Gruppe mit 5,8 % vs. 23,5 % (Odds Ratio 5; 95 %-CI 1,57–15,86; *p* = 0,003) gezeigt. Der Effekt verlief sich jedoch nach 12–24 Monaten, bei im Verlauf deutlich geringerer Fallzahl (*N* = 42 nach 24 Monaten vs. 137 bei Baseline). Die übrigen FCSRT-Scores (TR, TFR, DTR) konnten zwar auch zu allen Zeitpunkten eine Überlegenheit des HA-PCI-Arms bezüglich des neurokognitiven Funktionserhalts zeigen, jedoch nur vereinzelt oberhalb des definierten Signifikanzniveaus. Während die Autoren die Knappheit an Daten nach 24 Monaten als Limitation der Studie einräumen, gehen sie leider nicht näher auf einen inhaltlichen Vergleich der unterschiedlichen FCSRT-Scores ein. Es wird lediglich geäußert, dass der DFR-Score die größte Vergleichbarkeit zum Delayed-recall-Score des HVLT habe.

Im Hinblick auf die Lebensqualität konnte zwischen beiden Behandlungsarmen kein signifikanter Unterschied festgestellt werden. Vor allem die Cognitive-functioning-Skala des QLQ-C30, welche im „data supplement“ aufgeführt ist, sollte eine für den Patienten im Alltag spürbare – und somit klinisch relevante – Beeinträchtigung der Neurokognition erfassen können. Der vermutete „benefit“ der Hippocampusschonung könnte zu gering sein, um hier ein signifikantes Ergebnis hervorzubringen. Grundlegend wirft die vorliegende Arbeit also vielmehr die Frage auf, welche Methoden und Instrumente für die Messung der Neurokognition im untersuchten Patientenkollektiv tatsächlich geeignet sind, als dass sie eine Überlegenheit der Hippocampusschonung belegen könnte.

## Fazit

Unserer Meinung nach bleibt ein für die Patienten spürbarer klinischer „benefit“ durch die Hippocampusschonung während einer PCI letztlich weiterhin fraglich. Die Arbeit zeigt aber wie zuletzt auch die Daten von Belderbos et al., dass die Hippocampusschonung sicher durchführbar ist und keine erhöhte Inzidenz von Hirnmetastasen oder kein schlechteres Gesamtüberleben befürchtet werden muss. Gerade bei jungen Patienten ohne vorbestehende neurokognitive Einschränkungen lassen sich kaum valide Argumente gegen eine Hippocampusschonung finden, sodass sie auf Patientenwunsch mit gutem Gewissen angeboten werden kann. Der schwer quantifizierbare Einfluss auf die Neurokognition – und damit die klinische Relevanz der Schonung – wird wohl weiterhin Gegenstand der Forschung bleiben.

*Fabian Schunn und Stefan Koerber, Heidelberg*
